# Clinical Personality Patterns in Alcohol Use Disorder: A Study Focused on Sex Differences

**DOI:** 10.3390/jcm14145062

**Published:** 2025-07-17

**Authors:** Armando L. Morera-Fumero, Maria Natividad García-Gómez, Alejandro Jiménez-Sosa

**Affiliations:** 1 Department of Internal Medicine, Dermatology and Psychiatry, Faculty of Medicine, University of La Laguna, 38200 Santa Cruz de Tenerife, Spain; 2 San Miguel Addiction Association, 38006 Santa Cruz de Tenerife, Spain; marujagarciagomez@sanmigueladicciones.org; 3 Research Unit, University Hospital Complex of the Canary Islands, 38320 Santa Cruz de Tenerife, Spain; ajimenezsosa@gmail.com

**Keywords:** alcohol use disorder, personality disorders, sex differences, MCMI-III, personality disorder clusters

## Abstract

**Background:** Research on sex differences in personality disorders profiles among individuals with Alcohol Use Disorder (AUD) remains limited. This study aimed to examine sex differences in personality disorders in AUD individuals attending to an outpatient alcohol and drugs treatment unit. **Methods:** Persons seeking alcohol detoxification treatment were assessed with the Millon Clinical Multiaxial Inventory-III (MCMI-III) after abstinence. Both dimensional trait scores and cluster personality disorders types distribution were analyzed. A total of 216 subjects, 114 women (53%) and 102 men (47%), participated in the study. **Results:** No sex differences were found for paranoid, schizoid or schizotypal traits scores of Cluster A types. Women exhibited higher scores on the Cluster B histrionic trait (48 ± 22 vs. 39 ± 23, *p* = 0.012), with no differences in antisocial, borderline, or narcissistic trait scores. Narcissistic personality disorder was more prevalent in men than women (44% vs. 20%, *p* = 0.012). Cluster C dependent (52 ± 24 vs. 46 ± 20, *p* = 0.025) and obsessive-compulsive (54 ± 20 vs. 43 ± 19, *p* = 0.012) traits scores were elevated in women, but only dependent personality disorder prevalence differed categorically (38% women vs. 15% men, *p* = 0.012). **Conclusions:** Employing both dimensional and cluster approaches enriches personality disorder research in AUD. Dependent personality disorder in Cluster C robustly differentiates sexes, while personality disorder patterns in Clusters A and B show minimal sex differences when both approaches are considered.

## 1. Introduction

Personality disorders (PDs) and substance use disorders (SUDs) frequently co-occur and its relationship is a two-way road [1]. Individuals experiencing PDs often turning to substances to cope with their symptoms, leading to SUD [2]. Conversely, substance use can exacerbate PDs and negatively impact relationships [1]. This comorbidity can complicate treatment and increase the severity and chronicity of both conditions [3].

Alcohol is the most used legal drug in the world. Despite its negative effects when consumed in excess, alcohol is widely accepted socially and is present in many cultures [4].

Alcohol use disorder (AUD) is characterized by a maladaptive pattern of alcohol consumption resulting in clinically significant impairment or distress [5]. AUD and mental disorders, including PDs, are commonly associated [6]. Lifetime prevalence of AUD in major mental disorders is 22.3% while lifetime prevalence in subjects with no mental disorder is 11.0% [7]. Epidemiological data indicate that approximately 28.6% of individuals with AUD meet criteria for at least one PD [8]. Estimates of PDs prevalence among AUD populations varies widely from 13.9% [9] to over 48% in some clinical samples [10].

The study of personality disorders in AUD subjects have been carried out using different instrument to evaluate personality such as the Multiphasic Minnesota Personality Inventory (MMPI) [11], the NEO personality inventory [12], the Eysenck Personality Questionnaire (EPQ) [13], the Structured Clinical Interview for DSM Personality Disorders (SCID) [10] and the Cloninger Temperament and Character Inventory [14] just to name some of them Instruments such as the NEO Personality Inventory and the Eysenck Personality Questionnaire (EPQ) are not diagnostic tools for personality disorders. However, their mention is justified by the fact that they have been used indirectly, with the expectation that extreme scores on these questionnaires could suggest a presumptive diagnosis of personality disorder in those previous studies that explored the relationship between personality and AUD.

The Millon Clinical Multiaxial Inventory (MCMI) distinguishes personality pathology from clinical symptoms and aligns its scales with the DSM-5 PDs classification, grouping clinical personality types into three clusters: A (schizoid, schizotypal, paranoid), B (borderline, histrionic, narcissistic, antisocial), and C (dependent, obsessive-compulsive, avoidant) [15]. The use of the MCMI-III has been decisive because it is highly valid in clinical work with patients. On one hand, it provides us with information about PDs, and on the other, in Axis I, and in Axis II for PDs the MCMI-III gives us scores on the clinical syndromes they present. The MCMI has been used in the evaluation of the AUD as well as other drug use disorder [16,17,18].

Recent research highlights significant gaps in the reporting and analysis of sex and gender in randomized controlled trials (RCTs) [19]. This study found that while 98% of RCTs described the demographic composition of participants by sex, only 6% conducted subgroup analyses across sex, and a mere 4% reported sex-disaggregated data [19]. This suggests a critical need for more nuanced approaches to understanding how sex and gender influence health outcomes, particularly in conditions like AUD and PDs.

Despite the well-documented prevalence of AUD in both men and women, gender-based approaches are often overlooked in research and treatment [20,21]. Several studies [22,23,24] have emphasized the importance of considering gender differences in AUD, yet this perspective remains underutilized in clinical practice and research design.

Sex differences in PDs are also well-established. Avoidant, Dependent, and Paranoid PDs are significantly more frequent in women, while Antisocial PD is more common in men women [8]. These findings underscore the need for gender-sensitive diagnostic and therapeutic strategies in mental health care. Similarly, AUD exhibits notable gender differences. According to a one-year research, men have a 12-month prevalence of AUD of 14.1%, compared to 5.3% in women [25]. Despite these disparities, there is a paucity of research that simultaneously examines sex differences and personality disorders in AUD. This gap in the literature limits our understanding of how these factors interact and influence the course and treatment of AUD.

This study aims to investigate sex differences in PDs among AUD persons, using the MCMI-III, in a clinical sample requiring treatment.

## 2. Materials and Methods

### 2.1. Participants

Individuals requesting treatment for alcohol consumption problems at the Santa Cruz de Tenerife (Canary Islands, Spain) San Miguel Clinic of Drug Dependence and the Female Alcoholism Unit took part in the study. The sample was recruited between 2016 and 2021. Persons meeting DSM-5 diagnostic criteria for AUD took part in the study. AUD participants had a clinical picture between moderate and severe (>4 symptoms). The selection criteria were age between 18 and 65 years old and fluency in Spanish. The only exclusion criteria was the presence of cognitive impairment evaluated during the clinical assessment. Sex identity was self-reported.

### 2.2. Personality Disorders Assessment

The Millon Clinical Multiaxial Inventory-III (MCMI-III) is a self-administered clinical inventory composed of 175 items with dichotomous (true/false) response, designed to assess personality disorders patterns and clinical syndromes in adult population. The MCMI-III includes the 10 basic personality disorders pattern scales aligned with DSM-5 PDs [26]. It is structured in different scales based on the styles and traits defined in Millon’s model and are grouped into three major clusters:

Cluster A: Eccentric or bizarre personalities: Paranoid, Schizoid, Schizotypal;

Cluster B: Dramatic, emotional or erratic personalities: Histrionic, Narcissistic, Antisocial, Borderline;

Cluster C: Anxious or fearful personalities: Avoidant, Dependent, Obsessive-compulsive.

Each scale provides a Base Rate (BR), score designed to facilitate standardized clinical interpretation. The full BR ranges from 0 to 115, with the following cut-off points:

BR < 60: Not clinically significant;

BR 60–74: Personality traits present, without meeting criteria for disorder;

BR 75–84: Suggestive of presence of disorder (clinical level);

BR ≥ 85: Clear presence of disorder, clinically significant.

In this research, we analyzed the dimensional approach to personality disorder (trait scores) as well as the categorical approach to personality disorders (distribution of personality disorder types in each cluster).

### 2.3. Study Protocol

The study was conducted in accordance with the ethical principles of the Declaration of Helsinki and was approved by our Institutional Review Board. Persons requesting treatment for AUD took part in the study. All individuals signed a therapeutic contract where they agreed to share their data and clinical information in any research developed by the institution. Biopsychosocial information was collected through the interdisciplinary clinical history. Variables such as employment and educational situation, history of substance use (main substance, age of onset, frequency), personal and family medical and psychiatric history, social and economic consequences derived from use, as well as the family support network were evaluated. The therapeutic team (physician, psychologist, and social worker) collected the data. The MCMI-III was filled by the participants in a situation of total alcohol abstinence after detoxification. To monitor individuals’ withdrawal status, routine urine drug screenings were performed.

### 2.4. Statistical Analysis

Continuous variables are presented as means ± standard deviations, and categorical variables as frequencies and percentages. We applied two complementary statistical approaches to examine sex differences in personality. First, we used Student’s *t*-tests to compare mean scores on each MCMI-III personality scale between men and women, which allowed us to assess average differences in trait expression.

Second, to provide a clinically meaningful interpretation, we classified participants based on Millon’s base rate (BR) thresholds into three categories: low (BR: 60–74), moderate (BR: 75–84), and high/clinically significant (BR: 85–115). We then compared the distribution of individuals across these categories using chi-square test or Fisher’s exact test, depending on expected cell sizes. Specifically, chi-square tests were used when all expected cell counts were ≥5, while Fisher’s exact tests were applied when this assumption was violated. Pairwise comparisons between groups were also performed using the same tests.

To control for multiple comparisons and reduce the risk of Type I error, we applied the Benjamini–Hochberg procedure [27] to control the false discovery rate. All tests were two-tailed, and *p* < 0.05 was considered statistically significant. Analyses were conducted using IBM SPSS Statistics for Windows, Version 25.0 (IBM Corp., Armonk, NY, USA).

## 3. Results

### 3.1. Sample Characteristics

Two hundred and twenty-three persons living in the community participated in the study, of whom seven were excluded due to invalid MCMI-III results. The seven excluded individuals had a validity base rate of 1, that means that the subject’s response pattern is unreliable and uninterpretable and should not be used. The final sample included 216 participants: 114 women (53%) and 102 men (47%). No participants identified outside the male/female binary.

### 3.2. Sociodemographic Differences by Sex

Table 1 presents the results of the sociodemographic comparison by sex.

No significant sex differences were observed in age, number of participants with children, employment situation, or educational level. Men were more frequently married or partnered (*p* = 0.013) while women were divorced/separated more often (*p* = 0.025). Men lived more frequently with the family of origin (*p* = 0.018) and after applying the Benjamini–Hochberg procedure, the significance of this comparison was still present.

### 3.3. Clinical Characteristics by Sex

Table 2 shows the comparison of clinical characteristics by sex.

Women asked for help significantly before men, so women had significantly fewer years of consumption than men (*p* = 0.001). There were no sex differences between women and men regarding the consumption pattern (regular versus occasional) and the type of treatment they received for detoxification (Outpatients Addiction Unit, Hospital Detoxification Unit, Residential Unit for Addiction Disorders). Women and men had a high percentage of family with antecedents of drugs consumption, being significantly higher in men than women (*p* = 0.02). With respect to the secondary use of drugs, men preferred to use more cocaine than women (*p* = 0.013) while women preferred to use more benzodiazepines than men (*p* = 0.005). The differences remained statistically significant after applying the Benjamini–Hochberg procedure.

### 3.4. Personality Trait Scores by Sex

Table 3 presents the comparison of the personality trait scores by sex.

Regarding Cluster A, no significant sex differences scores were found. In Cluster B, antisocial, borderline, and narcissistic trait scores did not differ between women and men. Women had significantly higher histrionic trait scores than men (*p* = 0.012). Cluster C dependent (*p* = 0.025) and obsessive-compulsive (*p* = 0.012) traits were higher in women. Avoidant trait did not differ between women and men. The differences remained statistically significant after applying the Benjamini–Hochberg procedure.

### 3.5. Personality Clusters Trait Type Distribution by Sex

Table 4 presents the comparison of personality traits levels distribution by sex.

No Cluster A personality differences in distribution percentage (paranoid, schizoid and schizotypal) were found by sex. Regarding Cluster B personality distribution by sex, no antisocial, borderline, or histrionic differences by sex were found. Cluster B, narcissistic personality disorder, was more prevalent in men than women (44% vs. 20%; *p* = 0.012). In the case of Cluster C, no sex differences in avoidant and obsessive-compulsive personality distribution were found. However, dependent PD was significantly more frequent in women than men (39% vs. 15%; *p* = 0.012). The differences remained statistically significant after applying the Benjamini–Hochberg procedure.

## 4. Discussion

To our knowledge, this is the first study to examine Alcohol Use Disorder (AUD) using the MCMI-III Clinical Personality Inventory, applying both a dimensional (trait scores) and categorical (suggestive of symptoms, indicative of abnormality, presence of abnormality) approach in a sex-balanced community sample. Our findings demonstrate that sex differences vary depending on the analytical approach: while trait scores revealed higher paranoid, histrionic, dependent, and obsessive-compulsive traits in women, only dependent Personality Disorder (PD) in women and narcissistic PD in men remained significantly different when applying categorical thresholds.

Regarding sex differences in secondary drug use, women with AUD reported higher benzodiazepine use than men, whereas men reported more cocaine use. These results align with clinical evidence suggesting that women with AUD are more likely to receive benzodiazepine prescriptions and misuse them, driven by self-medication for anxiety, prescription biases, and cross-dependence with alcohol—further compounded by higher psychiatric comorbidity [28]. Conversely, increased cocaine use among men with AUD is well documented [28,29]. Explanations include higher dopamine release and subjective reinforcement in men that may promote repeated and heavier use, while hormonal cycles in women may modulate use but do not offset the generally higher usage rates observed in men. Additionally, men are more likely to engage in polydrug use involving both alcohol and cocaine [28,29]. These findings have important implications for gender-informed treatment strategies that consider not only AUD but also sex-specific patterns of secondary substance use.

Clinically, we found that men had a longer history of alcohol use before seeking treatment, which could reflect delayed help-seeking behaviors, possibly influenced by gender norms that discourage acknowledging vulnerability. In contrast, women tended to seek help earlier, which may reflect greater self-awareness of the problem and of earlier-onset physical and psychological complications [30]. Alternatively, this difference may also stem from limited social support or inadequate referrals from primary care, consistent with studies highlighting the “invisibility” of women with AUD in healthcare settings [31].

In Cluster A, we found no significant sex differences in paranoid, schizoid, or schizotypal traits or in PD prevalence. Our results are consistent with recent epidemiological findings [32,33]. While some prior studies found no sex differences in Cluster A [34,35], others reported a higher frequency of paranoid, schizoid, or schizotypal PDs in men [36]. The variation in tools used to assess personality disorders (e.g., MCMI-I, MCMI-III, International Personality Disorder Examination) may account for these discrepancies.

In Cluster B, antisocial trait scores and PD prevalence were similar in both sexes, in line with some of the literature [33,35,36]. We also found no sex differences in borderline traits or PD prevalence, which agrees with other studies [36,37]. However, some studies have reported higher borderline traits or PD prevalence in women [34]. Women in our sample had higher histrionic trait scores but not a higher prevalence of histrionic PD, aligning with some previous findings [38], while contrasting with others that reported no sex differences [33,36,37] or even a higher prevalence in men [35]. Our finding of greater narcissistic PD prevalence in men aligns with most contemporary studies [36,39], although some have reported no sex differences [33].

In Cluster C, women scored higher on dependent and obsessive-compulsive traits, though only dependent PD prevalence reached statistical significance. These results mirror epidemiological patterns indicating higher dependent PD prevalence among women [40]. Overall, women with AUD showed a higher prevalence of Cluster C traits (fearful and anxious personalities), whereas men exhibited more narcissistic and antisocial traits. This suggests divergent relational and emotional regulation styles: men are more likely to externalize distress, while women tend to internalize it, potentially affecting the chronicity of the disorder and the likelihood of seeking help [41]. The absence of sex differences in avoidant traits may be due to the difficulty of detecting this personality disorder pattern in the context of active alcohol use, as addictive behaviors can mask or substitute typical avoidance strategies. Avoidant PD is among the most prevalent among individuals with alcohol dependence, with a reported comorbidity rate of 20% [42]. However, its manifestation may be attenuated or obscured by active drinking, complicating diagnosis.

Age also influences personality traits [43], with evidence indicating that personality tends to stabilize in middle and later adulthood [44]. Our sample, with a mean age around 50 and no significant age differences between sexes, is likely in the stable phase of the age–personality relationship. Therefore, age is unlikely to have impact on our results.

Another factor that may explain discrepancies relates to the ongoing debate between self-report and clinician-reported assessments of personality disorders. Self-report tools offer standardization and minimize clinician bias. Research highlights that self-report measures may be less prone to defensive responses than clinical interviews, as they allow for private disclosure of sensitive experiences, reducing stigma-related inhibition [45]. However, despite the advantages of instruments like the MCMI-III (e.g., feasibility, cost-efficiency, access to subjective experience), their limitations must be acknowledged. In line with Zimmerman [46], we emphasize the need to develop and apply diverse assessment methods. A key concern is reporting bias, as individuals with specific personality disorder traits may under- or over-report symptoms due to limited insight, denial, social desirability, or defensiveness. For example, individuals with narcissistic or antisocial traits may minimize maladaptive behaviors, whereas those with dependent or avoidant traits may exaggerate their distress [47]. These biases can affect diagnostic accuracy and prevalence estimates. Therefore, future research should incorporate multimethod assessments, including clinician-administered interviews and collateral informant reports, to improve diagnostic validity and reliability.

The two statistical approaches we used—mean score comparisons and categorical PD analysis—provide complementary insights. Mean-based comparisons reflect continuous variation and are sensitive to subtle sex differences, although these may lack clinical relevance. In contrast, categorical analyses identify clinically significant thresholds (e.g., BR: 85–115), highlighting individuals who meet criteria for pathological personality traits. Importantly, these methods may yield different conclusions. For example, a non-significant difference in mean scores may coexist with a significant difference in the proportion of individuals meeting diagnostic thresholds. This discrepancy underscores the value of using both approaches. Trait score analyses help characterize general trends, while categorical data inform clinical interpretation and real-world implications. Together, they offer a more nuanced understanding of sex-related differences in personality among individuals with AUD and guide both assessment and treatment strategies.

## 5. Limitations

The cross-sectional design limits causal inferences. Participants were recruited from a single center, which may limit generalizability. The sample consisted of individuals receiving healthcare services for addiction, representing only a subset of the broader AUD population. Another limitation is the underrepresentation of men relative to the epidemiologically established 75:25 male-to-female ratio among individuals with AUD. This sampling imbalance may have introduced selection bias, potentially affecting the generalizability of our findings and the observed sex differences in personality disorder profiles. Future research should strive to recruit samples that more accurately reflect the sex distribution of the AUD population to minimize bias and improve external validity.

## 6. Conclusions

This study provides novel insights into sex-based personality differences in individuals with AUD, using both dimensional and categorical frameworks via the MCMI-III. Women scored higher on paranoid, histrionic, dependent, and obsessive-compulsive traits, though only dependent PD was clinically significant. Men exhibited greater narcissistic PD prevalence. These differences underscore the importance of including sex as a key variable in psychopathological assessment and intervention design.

Integrating dimensional and categorical perspectives allows for a nuanced understanding of personality–AUD interactions, enhancing diagnostic precision and informing tailored treatment strategies. Clinically, this supports developing interventions that address interpersonal dependency in women and narcissistic dynamics in men to improve engagement and outcomes. Gender-specific patterns in secondary substance use further highlight the need for personalized care.

We recommend longitudinal, multicenter studies to track personality profile changes over treatment and their relationship to outcomes, incorporating cultural and intersectional perspectives to enhance generalizability.

## Figures and Tables

**Table 1:** Comparison of sociodemographic characteristics by sex. Continuous variables are presented as mean ± SD. Categorical variables are presented as absolute number N and percentage (%).

Variables	Women	Men	*p* Value	*p* Value Subclass
Age	46 ± 11	48 ± 10	0.19	
*Marital status*				
Married/partner	32 (28%)	44 (43%)	0.01	0.03
Divorced/separated	48 (43%)	28 (28%)		0.025
Single	29 (25%)	30 (29%)		0.50
Widow	5 (4%)	0 (0%)		0.06
Participants with children	76 (67%)	64 (63%)	0.55	
*Living with*				
Alone	44 (38%)	25 (25%)	0.04	0.27
Own family	48 (42%)	42 (41%)		0.89
Family of origin	20 (18%)	32 (31%)		0.018
With friends	1 (1%)	3 (3%)		0.99
Institution	1 (1%)	0 (0%)		0.99
*Educational level*				
Primary	10 (9%)	5 (5%)	0.13	
High school	37 (32%)	44 (44%)		
Professional training	33 (29%)	30 (29%)		
University	32 (28%)	23 (23%)		
Employment situation				
Active/working	53 (47%)	52 (41%)	0.61	
Unemployed	36 (32%)	26 (26%)		
Pensioner	25 (22%)	24 (24%)		

**Table 2:** Comparison of clinical characteristics by sex. Continuous variables are presented as mean ± SD. Categorical variables are presented as absolute number N and percentage (%).

Variables	Women	Men	*p* Value
Years of consumption	15 ± 7	21 ± 7	0.001
*Consumption pattern*			0.62
Regular	98 (86%)	90 (88%)	
Occasional	16 (14%)	12 (12%)	
Family member with toxic antecedents	63 (55%)	72 (71%)	0.02
*Secondary drug use*			0.001
Benzodiazepines	14 (12%)	2 (2%)	0.005
Cannabis	10 (9%)	4 (4%)	0.26
Cocaine	24 (21%)	37 (36%)	0.013
*Treatment type*			0.94
Outpatient Unit	55 (48%)	51 (50%)	
Hospital Detoxification Unit	37 (33%)	33 (32%)	
Residential Unit for Addiction	22 (19%)	18 (19%)	

**Table 3:** Comparison of personality trait scores by sex. Data are given as mean ± SD.

Personality Clusters	Personality Traits	Women	Men	*p* Uncorrected	*p* Corrected
**Cluster A** Eccentric, Odd	Paranoid	60 ± 20	53 ± 22	0.05	Not Significant
	Schizoid	56 ± 18	53 ± 19	0.36	Not Significant
	Schizotypal	48 ± 21	42 ± 26	0.07	Not Significant
**Cluster B** Erratic, Dramatic	Antisocial	64 ± 13	65 ± 14	0.64	Not Significant
	Borderline	54 ± 19	54 ± 18	0.39	Not Significant
	Histrionic	48 ± 22	39 ± 23	0.001	0.012
	Narcissistic	60 ± 16	64 ± 17	0.13	Not Significant
**Cluster C** Fearful, Anxious	Avoidant	46 ± 25	43 ± 23	0.33	Not Significant
	Dependent	52 ± 24	46 ± 20	0.002	0.025
	Obsessive-compulsive	54 ± 20	43 ± 19	0.001	0.012

**Table 4:** Distribution of personality trait levels by sex. Categorical variables are presented as absolute number N and percentage (%).

Personality Cluster	Personality Trait	Level	Women	Men	*p* Uncorrected	*p* Corrected
**Cluster A,** Eccentric, Odd	Paranoid	85–115	5 (6)	2 (4)	0.30	Not Significant
		75–84	10 (12)	12 (21)		
		60–74	71 (83)	43 (75)		
	Schizoid	85–115	2 (3)	2 (4)	0.99	Not Significant
		75–84	5 (7)	4 (8)		
		60–74	61 (90)	44 (88)		
	Schizotypal	85–115	0 (0)	1 (2)	0.21	Not Significant
		75–84	0 (0)	1 (2)		
		60–74	51 (100)	41 (95)		
**Cluster B,** Erratic, Dramatic	Antisocial	*85–115*	5 (5)	6 (8)	0.64	Not Significant
		*75–84*	11 (12)	12 (15)		
		*60–74*	76 (83)	61 (77)		
	Borderline	*85–115*	4 (7)	0 (0)	0.12	Not Significant
		*75–84*	3 (6)	3 (5)		
		*60–74*	48 (87)	53 (95)		
	Histrionic	*85–115*	1 (2)	3 (16)	0.07	Not Significant
		*75–84*	7 (16)	5 (26)		
		*60–74*	36 (93)	11 (92)		
	Narcissistic	*85–115*	6 (9)	10 (16)	0.013	0.012
		*75–84*	8 (11)	17 (28)		
		*60–74*	56 (80)	34 (56)		
**Cluster C,** Fearful, Anxious	Avoidant	*85–115*	2 (5)	1 (3)	0.30	Not Significant
		*75–84*	14 (34)	5 (16)		
		*60–74*	25 (61)	26 (81)		
	Dependent	*85–115*	7 (13)	0 (0)	0.013	0.012
		*75–84*	13 (25)	5 (15)		
		*60–74*	33 (62	28 (85)		
	Obsessive-compulsive	*85–115*	3 (5)	1 (6)	0.092	Not Significant
		*75–84*	6 (10)	6 (33)		
		*60–74*	51 (85)	11 (61)		

## Data Availability

The data presented in this study are available on request from the corresponding author.
